# The Unmet Needs of Caregiving Skills, Support, Emotions, and Finances of Stroke Caregivers: A Multicenter Study

**DOI:** 10.7759/cureus.44346

**Published:** 2023-08-29

**Authors:** Nor Shahrina Mohd Zawawi, Noor Azah Abd Aziz, Rebecca Fisher, Kartini Ahmad, Mohd Azahadi Omar, Marion F Walker

**Affiliations:** 1 Department of Medical Rehabilitation Services, Hospital Canselor Tuanku Muhriz Universiti Kebangsaan Malaysia, Kuala Lumpur, MYS; 2 Faculty of Medicine, National University of Malaysia (Universiti Kebangsaan Malaysia), Kuala Lumpur, MYS; 3 Faculty of Medicine & Health Sciences, University of Nottingham, Nottingham, GBR; 4 Faculty of Health Sciences, National University of Malaysia (Universiti Kebangsaan Malaysia), Kuala Lumpur, MYS; 5 Sector for Biostatistics and Data Repository, National Institutes of Health, Shah Alam, MYS

**Keywords:** malaysia, caregiver, long-term care, unmet needs, stroke

## Abstract

​​​​​​Background

Informal stroke caregivers in Malaysia play an important role in supporting stroke survivors following acute care. Nevertheless, there is a lack of available data to inform the sufficiency of help and resources available to address the needs of local stroke caregivers. This study aimed to determine the unmet needs in caregiving skills, support, emotions, and finances as well as the associated factors of stroke caregivers in Malaysia.

Methodology

This multicenter, cross-sectional study used a self-administered survey developed and validated for the Malaysian population. It was prepared in paper-based and web-based formats, and it was distributed via direct contact with the respondents, post, and email. Respondents were recruited from different sites in Malaysia. In this study, unmet needs were defined as “help that was needed more or was not provided to assist caregivers and address their specific needs.” This article only presents the quantitative data of this study. Data were analyzed using descriptive analysis and logistic regression to determine factors associated with unmet needs.

Results

Almost all study respondents (91%) reported having unmet needs. Unmet needs ranged from 1 to 10, while the mean unmet needs was 5. The highest unmet need was related to financial support (72.5%), followed by support from professionals to address their own needs (59.2%), skills to care for stroke survivors, i.e., skills in caring for stroke survivors with their daily activities (57.9%), and skills in supporting stroke survivors to perform rehabilitation at home (53.1%). The lowest unmet need was related to support in transporting stroke survivors from place to place (45.3%). Additionally, this study did not identify an association between the reported unmet needs and gender, age, ethnicity, duration of caregiving, and site of participation.

Conclusions

This study reported a range of unmet needs perceived by stroke caregivers in Malaysia. Further research is warranted to understand the gaps in supporting local stroke caregivers to inform future post-stroke support and services in the country.

## Introduction

Stroke is a major public health issue in Malaysia and was ranked as the second main cause of death and disability in 2019 [1]. The incidence of stroke in Malaysia increased by 5% from 2008 to 2016 and is more common in men of younger age groups (35-39 years old) [2]. The rising incidence of stroke can be attributed to the growing prevalence of non-communicable diseases, such as diabetes mellitus, hypertension, hyperlipidemia, and obesity, in the country [3].

Malaysia has made considerable progress in stroke care in recent years. With an earlier focus on acute stroke care and stroke risk management [4], there have been initiatives to advance pre-hospitalization care and post-stroke care, namely, with stroke in pregnant women and in older age groups [5]. Within the older age group, guidelines emphasize the importance of discharge planning, joint decision-making of care between multidisciplinary rehabilitation team members, stroke survivors and their caregivers, and end-of-life care [5]. In addition, the concept of long-term stroke care has been introduced in Malaysia for more than two decades which emphasizes the involvement of primary care services in providing post-stroke services beyond hospital discharge [6]. Nevertheless, the provision of post-stroke services in Malaysia is challenged by a lack of multidisciplinary care and social support services [7]. These include the lack of availability, accessibility, and effective stroke rehabilitation systems within the community which contribute to the ineffectiveness of post-stroke care services [8]. Furthermore, the existence of stroke care and resources are largely focused on stroke survivors, but very little on caregivers. Moreover, although recommendations about stroke rehabilitation and education for caregivers have been made in the guidelines, they are not clear and require further elaboration to ensure effective implementation.

In Malaysia, the care of stroke survivors in the community is typically transferred to their caregivers. However, with the lack of clear guidelines, training, and support for stroke caregivers, they may not be able to execute the caregiving role effectively and, consequently, may carry a high caregiving burden, especially at the early stage of caregiving (i.e., within a month after stroke) [9]. In addition, stroke caregivers in Malaysia also report experiencing caregiving burden which is aggravated by financial challenges and poor social support [10]. The inadequacy of support may cause them to experience extreme challenges in managing what for many must be an unexpected life change, fulfilling different roles such as employment and attending to their own personal needs. Previous studies have reported that stroke caregivers in Malaysia require support to facilitate stroke survivors to perform religious activities [11], and they have a lack of knowledge about positioning stroke survivors properly [12]. A recent study reported that local stroke caregivers expressed needing information on stroke and stroke rehabilitation as well as psychological support [8].

Establishing post-stroke care that adopts survivor and caregiver-centered practice would require an understanding of the needs and sufficiency of current post-stroke help and support from the perspective of stroke survivors and their caregivers. This objective understanding would help guide any required change in stroke care policy and practice [13]. Therefore, this study aimed to determine the prevalence of unmet needs among stroke caregivers in Malaysia in terms of finances, caregiving skills, emotions, and support. The association of unmet needs and the characteristics of the caregivers was also examined to estimate their risk of having unmet needs. From these findings, we hope to inform the direction and prioritization of post-stroke care for stroke caregivers in the local setting.

## Materials and methods

Study design and site

**Figure 1:**
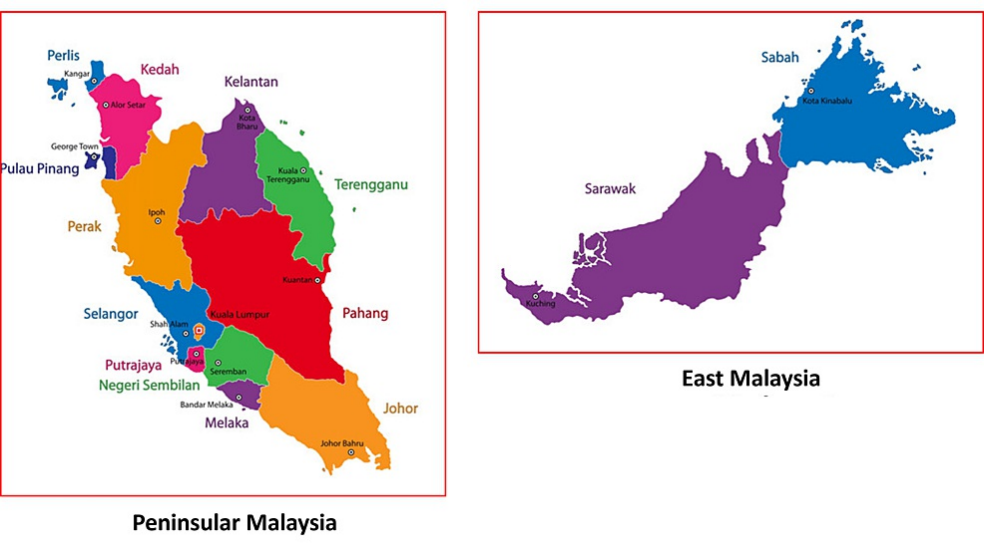
Map of Malaysia. Source of the original picture: https://www.vectorstock.com/royalty-free-vector/map-of-malaysia-vector-1607161

Malaysia is situated in the Southeast Asia region and is formed by Peninsular Malaysia and East Malaysia (Figure 1). This multicenter, cross-sectional study aimed to capture the unmet needs of stroke caregivers from different localities in Malaysia. Respondents were recruited from four sites, i.e., the National University of Malaysia Medical Centre (UKMMC), National Stroke Association of Malaysia (NASAM), Muar Health Information Centre, and Facebook pages (Stroke Caregiver Support Group Malaysia and Stroke Survivors Malaysia). Different sources were utilized to enhance the representativeness of survey responses from different regions of Malaysia. The site selection was based on knowledge of published stroke care centers and resources in Malaysia as well as access to the services.

Study design and survey development

The survey form for this study was developed based on a systematic narrative review [14] and interviews with stroke caregivers attending a stroke support group in Kuala Lumpur, Malaysia. The survey form consisted of 23 survey items. Questions about unmet needs were covered in 10 out of the 23 survey items, involving help with caregiving skills (three items), help with support in caregiving and non-caregiving tasks (four items), help with caregivers’ emotions (two items), and help with finances (two items). The remaining survey items were related to information provision, relationship status after stroke, and participation in stroke support groups. Content validation was performed with four care providers with vast experience in post-stroke care. Their opinions were sought to organize the identified items in a logical sequence and to remove duplicated items. They also examined the sentences in the survey form to ensure they were grammatically correct and precise in meaning. Additionally, face validation was performed with stroke caregivers through physical interviews. They were asked to provide feedback on the relevance of each survey item to meet the objective of this study (i.e., to capture the unmet needs of stroke caregivers). They were also asked about the clarity of the questions and pre-determined responses as well as the ease of feasibility. The survey form was formulated in two languages, Malay and English, to suit the multilingual preferences and language competencies of respondents. As the survey form was intended to be self-administered, each sentence in both versions was examined to ensure it was well understood by different ethnic groups in Malaysia. Additionally, the survey form was prepared in two formats, paper-based and web-based, to suit the preferences of the participants.

Sample calculation, study respondents, and data collection

This study adopted the formula for an infinite (unknown) population in calculating sample size as no previous studies have reported the prevalence of unmet needs of stroke caregivers in Malaysia. The expected prevalence was assumed to at 0.5 (50%), while the level of precision was estimated at 0.07 (7%); hence, the estimated sample size of respondents required for this study was 197.

The respondents of this study were the primary caregivers to stroke survivors receiving stroke care/support from the recruitment sites. They were the informal (unpaid) caregivers aged 18 years old and above, who were able to read or converse in English or Malay, and had been caring for stroke survivors for a minimum of six months to up to six years at the point of survey completion. Data collection was conducted over a seven-month period (December 2018 to June 2019). In this study, unmet needs focused on the perceived sufficiency of help with skills, support, emotions, and financial issues that respondents received in caring for stroke survivors. Additionally, respondents were also invited to write on the survey form any additional help that they felt was required. Respondents were also asked to provide their demographic details. All respondents received written information about the study. Respondents who completed the paper-based survey consented to their participation in writing. Likewise, respondents who completed the web-based survey acknowledged that the completion of the online survey would represent their consent to participate in this study. In this study, respondents who indicated their interest in participating via Facebook pages were emailed to verify their inclusion criteria. Those who met the inclusion criteria were sent the survey form, either via post or email, depending on their preference to participate. All survey forms were labeled before the distribution to enable tracking of the survey returns.

Definition of unmet needs and study variables

In this study, unmet needs referred to help that was still needed or not received by stroke caregivers in living life fully after a stroke. Unmet needs could be a result of many factors. Of note, the cause of the unmet needs was not investigated in this study. Only findings of the quantitative data of this study are presented in this article. Each survey item was analyzed individually. The dependent variable was the total of “help that was still needed” and “help that was not received,” while the independent variables were the respondents’ characteristics, i.e., gender, age, ethnicity, duration of caregiving, and center of recruitment. The selection of the independent variables was based on the reasons detailed below.

Gender

Female gender and stroke severity may contribute to a greater caregiving burden [14]. However, the unmet needs were reported to be comparable between stroke caregivers of all genders [15].

Age

The needs of younger stroke caregivers were different than those who were older [16]. Younger stroke caregivers may have higher demands to fulfill their caregiving role while keeping different roles to lead optimal living after stroke. In this study, older age refers to respondents aged 60 years and above, in accordance with Malaysia’s definition of the elderly [17].

Ethnicity

Ethnicity was found to influence the unmet needs of stroke caregivers [18]. The differences may be related to the cultural differences that influence the coping mechanisms after stroke, socioeconomic status, and the type of social support that they received to support living after stroke.

Duration of Caregiving

The changes in the needs of stroke caregivers vary at different times after stroke [19]. The variation could influence the unmet needs due to the changing priorities in adapting to care and living after a stroke.

Center

The availability and accessibility of post-stroke care services and resources are different across different regions and areas in Malaysia, which could lead to variations in the accessibility and availability of post-stroke care. Thus, in this study, geography was initially identified as the independent variable; however, due to the unequal distribution of respondents, geography was changed to center. In Malaysia, centers that deliver post-stroke care services focus largely on rehabilitation and medical care. Therefore, the examination of a center as a factor may contribute to understanding the association of different stroke service setups with types of unmet needs beyond rehabilitation and medical needs.

Statistical analysis

Data analysis was performed using the SPSS software version 26 (IBM Corp., Armonk, NY, USA). Following data entry into the software, missing data were identified. A deletion technique was applied to exclude missing responses from the analysis. Subsequently, frequency analysis was conducted to describe the respondents’ characteristics. A similar analysis was also used to identify the breakdown of the survey responses. Next, binary logistic regression was performed to assess the association between respondents’ characteristics and unmet needs. Before this analysis, responses that stated, “I have received some help, but require more” and “I did not receive help” were coded as “unmet” while other responses were coded as “other.” The regression analysis began with simple logistic regression to investigate the main effect of individual independent variables and the dependent variable. This analysis examined the likelihood of single respondents’ characteristics to the unmet needs. The characteristics were interpreted to significantly affect the unmet needs if the significance level was less than 0.05 (p < 0.05). Subsequently, multiple logistic regression was performed by examining all respondents’ characteristics in the analysis of unmet needs. Likewise, the association of the characteristics and unmet needs was considered significant if the significance level was less than 0.05 (p < 0.05).

Ethics and funding

This study was approved by the Research Ethics Committee, National University of Malaysia (approval number: JEP-2018-608) and funded by Nottingham University UK through a doctorate studentship.

## Results

Of the 585 survey forms that were distributed, only 272 (46.5%) survey forms were returned. Following an examination of the returned survey, 263 were eligible for analysis. The remaining survey forms were excluded as the demographic information was not provided. The majority of the respondents were from the central region of Peninsular Malaysia (Table 1). About two-thirds of respondents were women, and the majority were below 60 years old. About half were Malay and had been caring for stroke survivors for 24 months to 71 months. Overall, 56% of the respondents were spouses/partners of stroke survivors, while 34% were children of stroke survivors (Table 2). Furthermore, 91% of the respondents indicated a minimum of one unmet need. The range of the unmet needs was 1-10, with a mean of 5 (SD = 3).

**Table 1:** Distribution of respondents’ localities.

State/District	Total respondents
Peninsular Malaysia (Northern region)	
Kedah/Kulim	7
Penang/Georgetown	17
Perak/Taiping	1
Perak/Ipoh	11
Perak/Tanjung Malim	1
Peninsular Malaysia (Southern region)	
Negeri Sembilan/Seremban	2
Negeri Sembilan/Bahau	1
Malacca/Ayer Keroh	9
Johor/Muar	9
Johor/Kulai	1
Johor/Johor Bahru	28
Peninsular Malaysia (Eastern region)	
Pahang/Kuantan	4
Peninsular Malaysia (Central region)	
Federal Territory/Putrajaya	1
Federal Territory/Kuala Lumpur	136
Selangor/Shah Alam	1
Selangor/Petaling Jaya	24
East Malaysia	
Sabah/Kota Kinabalu	9
Sabah/Tawau	1

**Table 2:** Distribution of respondents’ characteristics (N = 263). UKMMC: National University of Malaysia Medical Centre; NASAM: National Stroke Association of Malaysia

	Total	%
Gender		
Women	174	66.2%
Men	89	33.8%
Age group		
60 years old and below	179	68.1%
More than 60 years old	69	26.2%
Missing response	15	5.7%
Ethnic group		
Malay	130	49.4%
Chinese	112	42.6%
Other	21	8.0%
Duration of caregiving		
Six months to 23 months	113	43.0%
24 months to 71 months	150	57.0%
Source of respondents		
UKMMC	137	52.1%
NASAM	103	39.2%
Other (Facebook pages and Muar Information Health Centre)	23	8.7%
Type of relationship		
Spouse/partners	146	55.7%
Parents	88	33.6%
Children/children-in-law	8	3.1%
Relatives	5	1.9%
Friends	15	5.7%
Missing response	1	0.4%

Unmet needs with skills, support, and emotions to care for stroke survivors

Overall, 50-60% of the respondents expressed having unmet needs, ranging from skills to care for stroke survivors, practical support to execute their own daily tasks, and personal needs. Regarding emotional support, 52.1% of the respondents reported having unmet needs from their informal resources to take a break from their caregiving roles, while 47.7% indicated having unmet professional support for their emotions (Table 3).

**Table 3:** Unmet needs with skills, support, and emotion to care for stroke survivors.

Type of skills	Percentage of respondents (%)			
	Received all the help needed	Received some help but need more	Did not receive help	No problem. Help was not needed
Category of help: Skills				
Skills in handling the daily activities of stroke survivors (N = 261)	22.6% (n = 59)	29.9% (n = 78)	28.0% (n = 73)	19.5% (n = 51)
		Total: 57.9% (n = 151)		
Skills in communicating with stroke survivors (N = 260)	16.2% (n = 42)	23.8% (n = 62)	28.5% (n = 74)	31.5% (n = 82)
		Total: 52.3% (n = 136)		
Skills in facilitating stroke survivors to continue rehabilitation at home (N = 260)	31.9% (n = 83)	29.6% (n = 77)	23.5% (n = 61)	15.0% (n = 39)
		Total: 53.1% (n = 138)		
Category of help: Support				
Support in caregiving tasks (N = 261)	22.2% (n = 58)	24.9% (n = 65)	22.6% (n = 59)	30.3% (n = 79)
		Total: 47.5% (n = 124)		
Support in executing daily tasks (N = 262)	19.5% (n = 51)	15.6% (n = 41)	35.5% (n = 93)	29.4% (n = 77)
		Total: 51.1% (n = 134)		
Informal support to transport stroke survivors (N = 263)	31.6% (n = 83)	19.4% (n = 51)	25.9% (n = 68)	23.2% (n = 61)
		Total: 45.3% (n = 119)		
Support from health professionals to address personal needs (N = 262)	17.9% (n = 47)	14.9% (n = 39)	44.3% (n = 116)	22.9% (n = 60)
		59.2% (n = 155)		
Category of help: Emotion				
Informal help to take a break from caregiving role (N = 262)	32.1% (n = 84)	30.9% (n = 81)	21.4% (n = 56)	15.6% (n = 41)
		52.3% (n = 137)		
Help from professionals to address emotions (N = 262)	8.0% (n = 21)	14.1% (n = 37)	33.6% (n = 88)	44.3% (n = 116)
		47.7% (n = 125)		

Unmet financial needs

Nearly three-quarters of the respondents (72.5%) reported having unmet financial needs, which is the highest unmet need reported in this study (Table 4). Among them, 171 respondents reported needing more financial help. The highest percentage related to bringing stroke survivors to their rehabilitation sessions/medical appointments (52.6%, n = 90), while helping to claim medical benefits/insurance was the lowest (39.8%, n = 68). Additionally, 44.4% (n = 76) required financial help for daily sustenance, while a similar percentage expressed that help was needed in searching for sources for financial help.

**Table 4:** Sufficiency of financial help received by the respondents to care for stroke survivors (N = 258).

Responses	Percentage of respondents (%)	
Received all the help that was needed	9.3% (n = 24)	
Received some help but need more	20.5% (n = 53)	Total: 72.5% (n = 187)
Did not receive help	51.9% (n = 134)	
Did not want help	1.2% (n = 3)	
Did not have financial problems	17.1% (n = 44)	

Factor associated with unmet needs

Following multiple logistic regression analysis, this study found that respondents from NASAM reported lower unmet financial needs compared to respondents from UKMMC (0.41, 95% CI = 0.21-0.82, p = 0.01). Unmet financial needs, however, were comparable between respondents recruited from NASAM and other centers (0.62, 95% CI = 0.21-1.83, p = 0.39). There were no other associations between unmet needs and gender, age, ethnicity, and duration of caregiving (Table 5).

**Table 5:** Associated factors of unmet financial needs by univariable and multiple logistic regression model. ^a^: Multiple logistic regression model was applied. Multicollinearity and interaction terms were checked and not found. Hosmer-Lemeshow test (p = 0.05), classification table (74.5%), and area under the receiver operating characteristic curve (0.68, CI = 0.60–0.75) were applied to check the model fitness. UKMMC: National University of Malaysia Medical Centre; NASAM: National Stroke Association of Malaysia

Variable	Simple logistic regression			Multiple logistic regression		
	Regression coefficient (B)	Odd ratio (95% CI)	P-value	Regression coefficient (B)	Odd ratio (95% CI)^a^	P-value
Gender						
Men	0	1		0	1	
Women	0.45	1.58 (0.89–2.78)	0.12	0.51	1.66 (0.90–3.08)	0.10
Age						
More than 60 years old	0	1		0	1	
60 years old and below	0.71	2.02 (1.11–3.69)	0.02	0.57	1.77 (0.93–3.38)	0.08
Ethnic						
Malay	0	1		0	1	
Chinese	-0.37	0.69 (0.39–1.21)	0.20	-0.10	0.91 (0.45–1.82)	0.78
Other	0.34	1.40 (0.44–4.47)	0.57	1.12	3.07 (0.61–15.61)	0.18
Duration of caregiving						
24–71 months	0	1		0	1	
6–23 months	0.37	1.44 (0.82–2.53)	0.20	0.38	1.45 (0.79–2.67)	0.23
Center						
UKMMC	0	1		0	1	
NASAM	-0.94	0.39 (0.22–0.71)	0.00	-0.88	0.41 (0.21–0.82)	0.01
Other	-0.37	0.69 (0.25–1.92)	0.48	-0.48	0.62 (0.21–1.83)	0.39

## Discussion

The results of this study support our assumptions that the scarcity of support and knowledge to support stroke caregivers has resulted in a wide range of unmet needs of stroke caregivers in Malaysia. In this study, respondents expressed the need for practical and emotional help to care for stroke survivors as well as to cope with the expected change in their roles in looking after their families. The most common unmet need was related to financial help. Over one-third of respondents reported insufficient help to address their personal needs, complete their daily tasks, and attend to their emotions. Additionally, over 50% of respondents had inadequate skills to care for and communicate with stroke survivors. The extensive unmet needs of stroke caregivers in Malaysia were in accordance with previous studies conducted by Denham and colleagues [20], who reported emotional, financial, and information-related unmet needs. Nevertheless, both studies found differences in the types and percentages of unmet needs, suggesting the importance of local knowledge about the unmet needs of stroke caregivers.

Caregivers may experience emotional resentment as a result of inadequate skills to care for stroke survivors and to attend to their own needs. Specifically, with skills to support stroke survivors with their rehabilitation process, the findings suggest a need to review the skills and knowledge of stroke rehabilitation personnel about working in partnership with stroke caregivers for effective rehabilitation, in addition to the setup and structure of the rehabilitation provision. The inequality in care distribution, workforce challenges, and service implementation may further contribute to the unmet rehabilitation-related needs, namely, with skills in communicating with stroke survivors [21].

Respondents in this study reported having higher unmet needs related to their own personal needs and non-caregiving tasks (36-44%) than caregiving matters (23-26%), suggesting they may have received some help to care for the survivors, particularly from their families. The lack of support to address their individual needs may increase their risk of developing depression, health problems, and psychosocial challenges [22]. The percentage of unmet emotional needs in this study was higher than in an Australian study [23]. These differences could be influenced by the differences in availability and access to psychological help in both countries, as well as the collectivist culture within the Asian family system such as “display a strong character during a stressful situation, and cover their emotions, as a way to protect the family pride” [24]. The findings suggested care providers need to be diligent in identifying the needs of stroke caregivers, for example, through conducting active screening or holding non-judgmental conversations.

The public healthcare services in Malaysia are highly subsidized for all Malaysians, with some services requiring citizens to pay out of their pocket [25]. Despite the nominal fee, the high percentage of unmet financial needs could be related to a disruption in their income [23], an increment in expenses associated with long-term care for stroke survivors [26], and financing the entire family [27]. In Malaysia, caregivers may seek financial help to care for stroke survivors from various government aids, examples include zakat (an obliged tax on Muslims), Baitulmal (an organization that is responsible for administering and managing the wealth of Muslims, as well as providing financial help for Muslim families), allowance from government social services and some non-government organizations; however, most of the financial help is minimal and below the country minimal wage (RM1500) [28]. Respondents of this study may also have had insufficient and unsustainable financial backups that they could rely on to meet the financial demands of their different living situations after stroke. These different factors may have contributed to this being the highest unmet need reported in this study.

In this study, gender, age, duration of caregiving, and ethnicity did not influence the unmet needs of stroke caregivers. It only found the center to be a predictor that influenced unmet financial needs among stroke caregivers. It is possible that stroke caregivers from NASAM, which is a private non-government organization that provides long-term stroke rehabilitation services, were able to meet expenses related to the rehabilitation fee and traveling cost to NASAM and have a better financial state compared to caregivers from other centers. In this study, information about the sociodemographics of caregivers was not collected; hence, we were unable to confirm these assumptions. Nevertheless, previous studies found that the provision of healthcare in Malaysia is challenged by inequity in resource distribution, accessibility, and financing [29]. Future studies with a higher number of respondents from different localities in Malaysia (rural and urban areas) and other characteristics such as socioeconomic, education of caregivers, and health literacy, in addition to the examined factors in this study, are necessary to further understand factors that would predispose stroke caregivers in Malaysia to have unmet needs.

Study strengths and limitations

This study used a survey derived from the evidence in the literature and the voices of stroke caregivers in Malaysia. These different resources ensured that the survey contained items that were important for the Malaysian population. The self-administered strategy also allowed respondents to reflect on their unmet needs on their own time and at an appropriate pace. This method may have reduced the confounding factors that may influence their response, e.g., in a rush, attending to other matters, or fear of being judged in the presence of a student researcher. In addition, the verification process of respondents who expressed their interest in participating in this study ensured that the reliability of the responses was not compromised. Nevertheless, many of the respondents in this study were from urban areas due to the locality of the two main centers used in this study, namely, UKMMC and NASAM. Therefore, the findings may not represent stroke caregivers from the rural areas of Malaysia as their needs may be different due to the limited availability and accessibility of stroke care, as well as support to return to the community. Additionally, with the low participation from the East Malaysia region (Sabah and Sarawak) (3.8%), the findings may need careful interpretation for stroke caregivers in these two states. With a health inequity between the East Malaysia region and Peninsular Malaysia [30], it is likely that the unmet needs would be different between these two regions. Finally, due to the nature of this self-administered survey, the completion and return of the survey relied on the interest of the respondents. This explained the discrepancies between the distributed questionnaires and the return rate of the survey response in this study, documented at a 46.5% return rate.

## Conclusions

In this study, a large percentage (91%) of stroke caregivers in Malaysia reported having unmet needs in carrying out different roles in living life after a stroke. The unmet needs were not associated with gender, age, duration of caregiving, ethnicity, and the center/medium that they sought out for support. The findings warrant targeted strategies to support stroke caregivers that may be required to further improve post-stroke care in the local setting. Further research that examines these unmet needs in relation to cultural factors, geographical variations, socioeconomic factors, and linguistic diversities is needed.
